# Extrachromosomal circular DNA in colorectal cancer: biogenesis, function and potential as therapeutic target

**DOI:** 10.1038/s41388-023-02640-7

**Published:** 2023-03-01

**Authors:** Yinnan Chen, Quanpeng Qiu, Junjun She, Jun Yu

**Affiliations:** 1 Center for Gut Microbiome Research, Med-X Institute, The First Affiliated Hospital of Xi’an Jiaotong University, Xi’an, Shaanxi China; 2 Department of High Talent, The First Affiliated Hospital of Xi’an Jiaotong University, Xi’an, Shaanxi China; 3 Department of General Surgery, The First Affiliated Hospital of Xi’an Jiaotong University, Xi’an, Shaanxi China; 4 Institute of Digestive Disease and Department of Medicine and Therapeutics, State Key Laboratory of Digestive Disease, Li Ka Shing Institute of Health Sciences, The Chinese University of Hong Kong, Hong Kong SAR, China

**Keywords:** Gastrointestinal cancer, Mechanisms of disease

## Abstract

Extrachromosomal circular DNA (ecDNA) has gained renewed interest since its discovery more than half a century ago, emerging as critical driver of tumor evolution. ecDNA is highly prevalent in many types of cancers, including colorectal cancer (CRC), which is one of the most deadly cancers worldwide. ecDNAs play an essential role in regulating oncogene expression, intratumor heterogeneity, and resistance to therapy independently of canonical chromosomal alterations in CRC. Furthermore, the existence of ecDNAs is attributed to the patient’s prognosis, since ecDNA-based oncogene amplification adversely affects clinical outcomes. Recent understanding of ecDNA put an extra layer of complexity in the pathogenesis of CRC. In this review, we will discuss the current understanding on mechanisms of biogenesis, and distinctive features of ecDNA in CRC. In addition, we will examine how ecDNAs mediate oncogene overexpression, gene regulation, and topological interactions with active chromatin, which facilitates genetic heterogeneity, accelerates CRC malignancy, and enhances rapid adaptation to therapy resistance. Finally, we will discuss the potential diagnostic and therapeutic implications of ecDNAs in CRC.

## Introduction

DNA is present in the form of chromosomes and is organized as chromatin architectures in eukaryotic. Extrachromosomal DNA (ecDNA) is a form of circular DNA element specifically found in the nuclei of cancer cells with a size range from dozens of kilobases to megabases [1]. ecDNA was first observed by Cox et al. described as double minus in 1965 [2], since then it has been detected in nearly half of the cancer types carrying oncogenes, including EGFR, ERBB2, MYC etc. in tumor cell lines, as well as clinical tumor samples [3–6]. Tumors containing ecDNA have been shown to have worse clinical outcomes compared to other forms of focal amplification [7–9]. Thus, ecDNA emerged as an oncogenic alteration in cancer genomes, which is highly associated with aggressive tumor behaviors [6, 7, 10]. ecDNA contains features as a potential vehicle of proto-oncogene amplification. Although with nucleosome structures, their circular structure is associated with an elevated transcription level compared to linear amplifications [11]. In addition, because of the lack of centromeres, oncogenes on ecDNA are randomly isolated after cell division and segregated into daughter cells unequally, which drives intratumoral heterogeneity by quickly increasing copy number under selection pressures [1, 3, 12, 13].

By genomic sequencing, bioinformatic approaches, and cytogenetic, ecDNA constitutes specific mechanisms for oncogene amplification and has been found in various types of cancer, including colorectal cancer (CRC) [1]. CRC accounts for about 10% of cancers diagnosed and relevant deaths worldwide annually [14]. It is the fourth most deadly cancer, with 900 000 deaths each year [15]. And intratumoral heterogeneity is one of the hallmarks of CRC, and most tumors contain cells with various degrees of differentiation [16]. A series of genome-wide studies of various CRC models have identified susceptibility genes associated with risk. However, most factors that could lead to heritability remain elusive [15].

Inheritance, variation, and selection are the basic principles of Darwinian organismal evolution that have been applied to explain the emergence, progress, and adaption of cancer cells [17, 18]. It is especially applicable to amplified oncogenes in CRC, whose cell-to-cell variability is high, even though the fitness advantage conferred. However, the mechanism that maintains heterogeneous oncogene amplification and the ability of CRC cells to rapidly adapt to various conditions, such as chemotherapy, by altering their genomes or the number of copies of the oncogenes has not been fully established. In addition, the delay in resistance of treatment by selection for drug resistance mutations that arises in a single or a small group of cancer cells raises questions about whether tumors are undergoing a genetic bottleneck [19]. The presence of ecDNA may help explain some of these controversial properties. Several studies have shown that ecDNA plays an essential role in the oncogenesis as well as drug resistance in CRC [20]. ecDNA is a circular chromatin particle without centromeres and telomeres; thus, it may rapidly accumulate in cancer cells through uneven inheritance, which offers a competitive advantage in response to pressures from anticancer drugs or unfavorable tumor microenvironment [21]. Given its structural complexity, prevalence, and oncogenic functions, there is renewed interest in the biology of this enigmatic molecule and its roles in the oncogenesis of CRC. In this review, we focus on recent progress in understanding the biogenesis as well as properties of ecDNA in CRC, which can provide the potential drug target and opportunities for therapeutic intervention for CRC.

## Biogenesis of ecDNA in colorectal cancer

### Formation of ecDNA in cancer

ecDNA commonly believed to originate from chromosomes [22]. The exact molecular mechanism of how ecDNA was formed remains elusive. Several models of ecDNA generation have been proposed, such as breakage-fusion-bridge (BFB) cycles, chromothripsis, the translocation-excision-deletion-amplification (TEDA) model, as well as episome model [3].

**Fig. 1:**
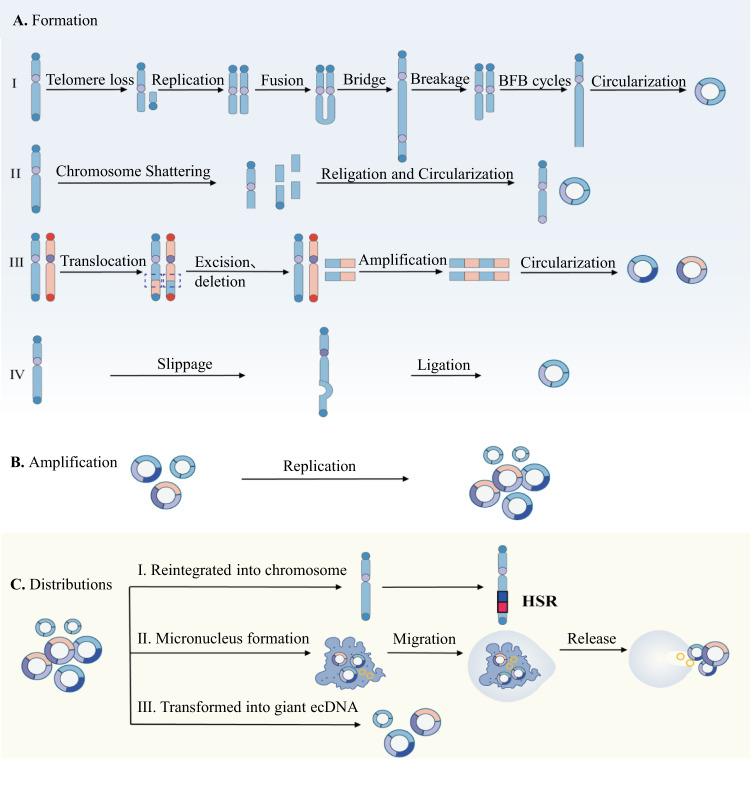
Biogenesis, amplification and distribution of ecDNA. **A** Formation of ecDNA. (I) Breakage-fusion-bridge (BFB) cycles. Loss of telomere because of genome instability, and the end of the missing telomere fuse with each other to form a chromosomal structure with two centromeres and a dicentric anaphase bridge. The fusion bridge is broken in the late stage of mitosis, keep the genes amplified and circularizing into ecDNA. (II) Chromothripsis model. When chromosomes are catastrophically broken, the DNA double-strand break into some DNA segments, which are randomly linked and cycled to form ecDNA during subsequent DNA repair. (III) Translocation-excision-deletion-amplification (TEDA) model. Segments between chromosomes translocation, DNA fragments between translocation breakpoints are prone to amplification, retention or deletion, and the deleted part is cyclized outside the chromosomes to form ecDNA. (IV) Episome model. Through the way of DNA slippage and R-loop, chromosomes form episomes during genetic recombination, ecDNA generated by cleavage and ligation. **B** Amplification of ecDNA replicates by rolling circle amplification. **C** ecDNA distributions. ecDNA can be subject to further clonal evolution, reintegrated into chromosomes, combined with other ecDNAs or eliminated by being trapped inside micronucleus.

The BFB cycle starts with the formation of anaphase bridges that connect the ends of sister chromatids or varies chromosomes, leading to arrays of genomic segments [23]. The newly formed chromosome is pulled in opposite directions during mitosis, which leads to the breakage of the chromosome and the triggering of the next cycle (Fig. 1A). BFB cycles can lead to chromosomal instability and aneuploidy, result in alterations in the copy number that contribute to the multiplicity of cancer initiation, progression, and therapeutic resistance, commonly found in CRC cells [24]. It has been shown in the plasmid-based model for analyzing how anaphase bridges break during mitosis in human CRC cell lines [25]. Since breakage occurs randomly, BFB cycles may generate various genomic aberrations, including both chromosomal as well as extrachromosomal gene amplifications. Meanwhile, colorectal adenocarcinoma is significantly enriched in somatic retrotranspositions, which can also initiate BFB cycles, leading to the amplification of oncogenes [26]. In short, the BFB cycle is a mechanism relevant for genomic instability [27], causes high levels of oncogenes amplification and telomeres can protect undamaged chromosomes, thus limiting repetitive BFB cycle events to a single chromosome arm [28, 29]. Studies in HCT116 colon carcinoma cells have also illuminated the genomic consequences of telomere dysfunction [30].

The shattering of one or more chromosomes and the production many DNA segments is called chromothripsis, which is characterized by genomic rearrangements and is generated in a catastrophic event [31, 32]. Constitutional chromothripsis has been reported to dampen APC expression and may result in a genetic predisposition to CRC [33]. Additionally, chromothripsis is a prevalent mechanism that drives structural rearrangements, and chromothripsis events drive CRC progression and metastasis [34–36]. With the help of the DNA repair system, these segments will be randomly religated to form complex rearrangement sequences or circularized into ecDNA with each other [32, 37] (Fig. 1A). The structural evolution of ecDNAs can go through several rounds of chromothripsis and incorporation of damaged DNA [37, 38]. Shoshani et al. reported that chromothripsis is an essential mechanism that accelerates the rearrangement and amplification of genomic DNA in ecDNA, thus enabling rapid acquisition of resistance to altered conditions in CRC [37]. These models are not exclusive, for instance, recent studies show that ecDNA may be initially produced by BFB cycles followed by chromothripsis [37]. The various coexisting amplicons in the same specimen indicate that ecDNAs may evolve into multi-fragment structures over time by a multistep process.

In the TEDA model ecDNAs are generated through amplified, deleted proto-oncogenes close to the chromosome translocation breakpoints and the fragile sites induced by hypoxia [39]. DNA fragments near the translocation breakpoint are usually less stable. Amplification events occur close to the translocation breakpoint and generate amplified chromosomal segments followed by circularizing to generate ecDNA (Fig. 1A). It could also be locally amplified and be retained into a chromosome to generate a uniformly and intensely staining segment named homogeneously staining region (HSR), which indicates amplification of a segment on a chromosome. The TEDA model can explain the co-amplification of genes from multiple chromosomes or fusion genes. Next, Carroll et al. reported that episomes were precursors of ecDNA and both of them can integrate into chromosomes [40]. In the episome model, a DNA fragment can be excised from a chromosome followed by circularization into circular DNA [38, 41] (Fig. 1A). It deletes sequences harboring a replication origin followed by repeated integration of chromosomal regions into the episome by recombination, leading to continuous enlargement of the episome. The episome model shows ecDNA can result from excision of circular DNA and enlarge by either replication or recombination [42]. Episomes can also self-replicate into multiple copies then reassemble to generate larger ecDNA [43].

### Compositions of ecDNA and amplification

Focal DNA amplifications in cancer include linear intrachromosomal and circular extrachromosomal forms [44, 45]. Comprehensive genomic characterization across various types of cancer has provided extensive catalogs of chromosomal rearrangements and vary greatly in focal chromosomal and extrachromosomal amplifications. (Fig. 1B) Clonal selection occurs when an oncogene or an oncogenic regulatory element affords the tumor cell advantage of proliferation or survival. More than 70 genomic regions have been reported to be amplified recurrently in cancer, and some of them are particularly important for the oncogenesis of CRC [46]. As an extrachromosomal circular chromatin, ecDNA can carry oncogene amplifications as a genetic entity. The oncogenes found most frequently in ecDNA include MYC, EGFR, MDM2, TERT, CDK4, ERBB2, SOX2, CCND1, E2F3, and CCNE1 [7]. Some oncogenes encode full-length open reading frames in ecDNA, whereas splicing variants or chimeric fusions are also found [4, 32]. This may result from circle-derived rearrangements, and multiple oncogenes may coamplified on the same ecDNA [47, 48]. In addition to those genes for protein coding, regulatory elements, such as enhancers, can also be amplified on ecDNA as the regulatory function of ecDNA in transcription [9, 49]. These enhancers are presumably formed through genome rearrangement by ecDNA [47]. As the molecule of ecDNA can span up to megabases, AmliconArchitect and Amplicon Reconstructor are generally applied to analyze the sequencing data of ecDNA [50, 51]. According to sequencing data from 117 cancer samples, the highly rearranged and heterogeneous patterns of ecDNA have been found in various types of cancer [50]. Furthermore, the structural patterns of ecDNA are heterogeneous. When comparing the primary tumor and its metastasis, the copy numbers and ecDNA segment boundaries were shifted in relapsed tumors [10, 48]. Their complexity and segments can be varied within a population of cancer cells derived from the same tumor [38]. New extrachromosomal amplified oncogenes can emerge, indicating a highly unstable and dynamic feature of ecDNA structures subject to clonal evolution [10].

### ecDNA distributions

After formation, ecDNA can undergo further clonal evolution, be reintegrated into chromosomes, combined with other ecDNAs, or be eliminated by being trapped inside micronuclei [4, 37, 52] (Fig. 1C). Due to the absence of centromeres, ecDNAs cannot be distributed evenly during the metaphase to anaphase stages of the cell cycle [53]. The sequence, size, and number of ecDNA molecules vary from cell to cell [1]. Jia et al. provided evidence of the distribution of amplicons in two different populations of ecDNA in CRC NCI-H716 cell line [54]. It was implicated that ecDNA plays a critical role in cancer heterogeneity in tumor cells and their progression. Uneven distribution together with the competitive advantage provided by the overexpressed oncogene will lead to the expansion of ecDNA-positive clones, and hundreds of ecDNA may be detected within a single nucleus [1, 6]. Thus, the frequency of ecDNA molecules will fluctuate in response to different circumstances [55]. Adaptive responses have been shown in tumor samples, where ecDNA-positive subclones shrink under specific treatment rapidly; however, recurrence occurs when stress is removed [6, 56]. The dynamic fluctuation of ecDNA levels could be particularly obvious under therapeutic stress conditions and may also be effective by unfavorable tumor microenvironments [57]. Additionally, epigenetic states have also been demonstrated to be associated with responses to selection pressure and can promote transient site-specific copy-number gains of gene locus, specifically when extrachromosomal [58, 59]. Eventually, ecDNAs are prone to accumulate mutations compared with chromosomal regions, which further promotes positive selection [60].

Reintegration of ecDNA into chromosomes that usually do not have their native locus can result in homogeneous staining regions (HSRs), coexisting with ecDNA in cells with similarly amplified segments [9, 32] (Fig. 1C). It can also be triggered by DNA damage and occurs at the free ends of DNA by double-strand breaks (DSB) [37]. In CRC cells, ecDNA induction and aggregation by DSB after breakage repair generate cytoplasmic micronuclei in the interphase, and micronuclear entrapment eliminates or transforms ecDNA into HSR [61]. If ecDNA elements did not segregate into a nucleus of the daughter cell after mitosis, they could be entrapped in micronuclei [62]. Mechanisms to ensure mitotic ecDNA distribution remain unclear. In the human CRC COLO 320DM cells, it has been reported that ecDNAs replicate early in the S phase, which is associated with active transcription [63, 64]. Transferring ecDNA molecules from the nuclear periphery to the center after the initiation of DNA replication indicates the specificity of the ecDNA replication machinery [65]. Shimizu et al. have shown that ecDNAs containing c-myc in CRC cell lines were localized and replicated in the nuclear periphery [66]. While in metaphase and during segregation, ecDNAs can bind to the telomeric regions of linear chromosomes [67]. Migration of ecDNA molecules into the same daughter cell after mitosis indicates that there are presumable post-replication bonds [68]. Several mechanisms for eliminating ecDNA have been proposed, such as physical exclusion from the cell, enzymatic degradation, and the elimination of micronucleated cells by cell death [69]. ecDNA can be removed from the nucleus by a micronucleation mechanism that initiates budding of the nuclear membrane during the S phase of mitosis in CRC cells [66] (Fig. 1C). Furthermore, Oobatake et al. show that in CRC cells, hydroxyurea induces random double-strand breakage leading to ecDNA aggregation in the S phase, and ecDNA is eliminated by micronuclear entrapment or transformed into giant ecDNA or HSR [61] (Fig. 1C). Meanwhile, the attenuation of homologous recombination activity decreased the number of amplified gene copies on ecDNA and increased the exclusion of micronuclei in CRC cells, which was also accompanied by cell cycle acceleration and methotrexate (MTX) sensitivity [70].

## Function of ecDNA for oncogenesis of colorectal cancer

### Oncogene overexpression and copy-number amplification

**Fig. 2:**
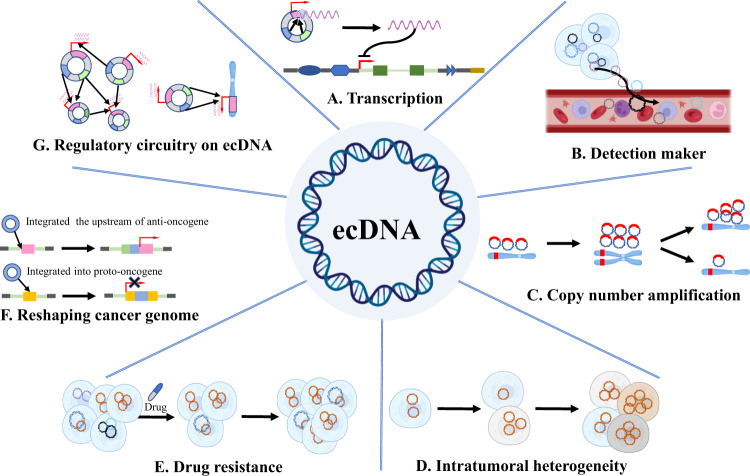
Functions of ecDNA for oncogenesis. **A** Transcription. ecDNA can transcribing into RNAs for protein translation or regulating gene expression. **B** Detection maker. Tumor cells release ecDNA into the blood circulation and thus serve as a tumor detection marker. **C** Copy-number amplification. Unequal division of ecDNA leads to rapid amplification of oncogenes carried on ecDNA compared to chromosomal DNA. **D** Intratumoral heterogeneity. Random assignment during ecDNA replication leads to tumor heterogeneity. **E** Drug resistance. Continuous amplification of ecDNA containing resistance genes leads to drug resistance in tumor cells. **F** Reshaping cancer genome. If ecDNA is integrated into the upstream of the proto-oncogene, it can enhance the expression of the proto-oncogene, and if integrated into the tumor suppressor gene, it will cause loss of tumor suppressor gene function. **G** Regulatory circuitry on ecDNA. Multiple ecDNA aggregates to form ecDNA hubs, the enhancers and promoters carried on ecDNA act on protein-coding genes, facilitating the transcription of oncogenes.

Human chromosomes are made up of DNA that is wound around nucleosomes controlled by regulators of DNA [71]. It regulates associations between genes and proteins to respond to intracellular and extracellular signals, and provides a control system that prevents incorrect interactions. Alterations in chromatin organization are associated with CRC, as well as with their resistance to drugs [72]. In CRC, ecDNA is associated with increased oncogene expression compared to linear amplicons, and the native chromosomal locus, even with copy-number normalization [1, 7] (Fig. 2A). This is partly driven by the lack of higher-order compaction of ecDNA in nucleosome arrays, which allows the interaction of transcription machinery with gene loci in CRC cell lines [47]. A significantly higher signal from ATAC-seq was observed indicating the association of ecDNAs with more accessible chromatin and increased transcriptional activity [7, 47]. In addition, the nanopore sequencing at single-molecule resolution showed that ecDNA chromatin is two times more accessible compared to homologous linear DNA chromatin, and 80% of ecDNA areas were accessible [73]. Multiple reasons could contribute to oncogene overexpression. Firstly, the circular structure of ecDNA is stable allowing highly increased DNA cis interactions compared with that of chromosomes. Secondly, ecDNA molecules are physically clustered in the nucleus and engage in intermolecular interactions. In addition, genome sequence rearrangements vastly changed the regulatory context of gene loci. Furthermore, the characteristic of ecDNA mobility makes it have the potential to become cell signaling molecules, which can produce short regulatory RNAs, such as microRNA and novel si-like RNA that modulate gene expression [74]. Compared with linear DNA, circular DNA is easier to cross the cell membrane into the circulatory system. The circular structures of ecDNA are relatively more stable and have a longer half-life in the blood circulation [75]. Currently, ecDNA has been detected in peripheral blood, and these facts indicate the potential of ecDNA in the application of tumor diagnosis and prognostic markers [76] (Fig. 2B). There could be additional mechanisms driving oncogene overexpression on ecDNA in CRC.

Gene copy-number amplification also played an essential role in CRC progression. ecDNA is associated with elevated oncogene expression compared to linear amplicons and the native chromosomal locus, which could be driven by gene copy-number amplication [1, 7, 77]. ecDNA can be generated derived from chromosomes, and at the same time, it can also be replicated independently of mitosis through rolling circle amplification, thereby achieving a rapid increase in gene copy number [78] (Fig. 2C). As we described above, due to the lack of centromeres, they can randomly distributed among the daughter nuclei during cell division [53]. The random segregation results in heterogeneity of cancer cells and those of which carry ecDNAs providing a fitness advantage under selection pressure (Fig. 2D). This feature of extrachromosomal oncogene amplification has been reported to lead to up to several hundreds of ecDNAs in a single CRC, as well as other types of cancer cells [1, 79–81]. This has been associated with rapid respond and adaptation to selective pressures, as well as increased of therapeutic resistance [6, 82] (Fig. 2E). A series of studies has shown aberrant amplified oncogenes on ecDNA in CRC cells such as HER-2, EGFR, c-myb, and myc [5, 83–85]. Meanwhile, in multiplying CRC cell lines Morales et al. show that overexpression of the DHFR gene and the presence of ecDNA plays an important role in resistance to MTX [20].

Furthermore, double-strand DNA breaks in ecDNA have been associated with aggregation of ecDNA molecules and formation of chromosomal tandem amplicons termed homogeneously staining regions (HSRs), suggesting that ecDNA clustering may also explain the formation of some chromosomal amplicons [61]. The reintegration of ecDNA may disrupt chromatin domains as well as cis-regulatory elements and thus regulate gene transcription. Integrated into the upstream of proto-oncogenes could enhance the expression of proto-oncogenes. Meanwhile, ecDNA fragments may also be inserted into suppressor genes, resulting in loss of tumor suppressor gene function and promoting tumor development [4] (Fig. 2F).

However, oncogene cis amplification or copy-number variation does not fully explain the high level of the expression oncogene observed in ecDNA-positive CRC cells. Recent studies raise the possibility that oncogenes expression from ecDNA is only partly determined by the increasing dosage of oncogene in ecDNA. The interplay between the local and distant enhancer, the extent of chromatin compaction and accessibility, the transcription factor affinity at the promoter binding sites and the DNA copy number, together determine the transcriptional level from ecDNA. It also suggests that there could be other potential mechanisms that regulate oncogenes expression on ecDNA different from traditional chromosomal regulation of gene expression.

### ecDNA hub as a novel structure for gene regulation

The three-dimensional structure of eukaryotic chromosomes is well organized at different length scales, including chromosome territories, compartments A and B, topologically associating domains (TADs), and chromatin loop [86]. The interaction between enhancer and promoter could span tens to hundreds of kilobases. Cis interactions between enhancers and target genes are usually found within TADs of the same DNA molecule. However, in CRC cells, can have up to hundreds of ecDNA clusters can be located in the nucleus; therefore, multiple ecDNA can interact with each other which forms cooperative interaction in the interphase named ecDNA hub [80] (Fig. 2G). ecDNA hubs represent a counterpart to chromosomes as units of genetic organization. Studies show that these ecDNA clusters are not randomly scattered, but localized in the nuclear periphery during the G1 phase and the M phase [65]. It is commonly believed that the nuclear periphery is transcriptionally repressed, whereas ecDNAs are transcriptionally active [87]. Therefore, its peripheral localization is counterintuitive and requires further study for its possible functional significance. Compared with linear arrays of genes activated by regulatory elements on the same DNA molecule, Huang et al. demonstrated that ecDNA hubs allow intermolecular gene activation by combinatorial promoter and enhancer elements in spatial proximity [80]. In addition, in CRC cells, ecDNA hubs condense during interphase but dispersed during mitosis, fundamentally distinguished from that of chromosomes [80]. ecDNA hubs are detected during mitosis with dynamic size alterations in various types of cancers, including CRC, indicating that they are not stable during DNA partition [55, 80, 88] (Fig. 2).

Transient transcriptional hubs play a critical role in gene transcription, and ecDNA hubs are mediated by protein-protein interactions [89, 90]. In CRC cells, the disruption of MYC ecDNA hub has been studied with suppression of the bromodomain and extraterminal (BET) proteins, but not by transcriptional inhibition with alpha-amanitin or 1,6-hexanediol [80]. This indicates that the ecDNA hubs are independent of the active transcription regions recognized by RNA polymerase II or intrinsically disordered regions sensitive to hexanediol [91]. BET proteins usually concentrate at the accessible DNA regions and can mediate enhancer-promoter communication [92]. Thus, ecDNA hubs could be involved in long-range looping to promote intermolecular chromatin interactions between ecDNAs. The formation of ecDNA hubs is closely associated with the oncogene transcription from each ecDNA molecule in CRC [55, 80]. The ecDNA hubs normally cluster 10–100 ecDNAs in proximity allowing intermolecular enhancer-promoter interactions [80]. Genes on linear chromosomes normally can be regulated by enhancer or other regulatory DNA elements on the same chromosome; however, ecDNA can engage enhancers from other ecDNA molecules within an ecDNA hub. As dozens of ecDNAs clustered together in various spatial configurations, it provides the possibility for ectopic interactions between enhancer and promoter that barely happen on linear chromosomes. Thus, it permits two molecular derived ecDNAs from two different chromosomes activate each other by enhancer and promoter contacts. Therefore, ecDNA hubs contribute to the long-range of enhancer usage and heterogeneity in oncogene activity among CRC cells [80].

The intermolecular interplay among ecDNAs with distinct enhancer elements provided the possibility for co-selection. Individual ecDNA contains functional enhancers that could activate oncogene expression and offer better fitness to CRC cells than the others. On the other hand, the co-selection could also occur at the ecDNAs hubs. Each ecDNA may not contain the complete set of gene regulate elements for oncogene activation, instead, they exist as a portion of an ecDNA hub that activates the target oncogene by promoting the interaction between regulatory elements and functional enhancers located on different DNA molecules. Meanwhile, the cooperative role of the ecDNA hub can increase the tolerance to mutations in each molecule [90]. The ecDNAs hub significantly expands the concept of CRC genetic heterogeneity, as cancer cells can carry ecDNAs with different oncogenes and diverse regulatory sequences, which may interact with each other or integrate into larger circular molecules. The randomness of offspring distribution, the fusion of ecDNA to form larger pieces, and the integration of ecDNA into chromosomes will affect the spatial clustering [81]. The ecDNA hub intrigues the concept that gaining survival advantages from clonal competition among cancer cells could take place through clonal cooperation among ecDNA molecules [3]. Remarkably increased the potential to drive diversity and accelerate evolution. Intermolecular gene activation could potentially distinguish between cellular physiology and CRC cells harboring ecDNA.

### Role of ecDNA in therapeutic sensitivity in colorectal cancer

The main reason for the failure of cancer treatments is the development of drug resistance. The amplification of the oncogene is a typical way of genome alterations, and it is a stepwise selection process for acquired drug resistance in which cancer cells become less sensitive to treatment through repeated cycles of cell death and proliferation with genomic instabilization and gene copy variations. And it is associated with tumor heterogeneity or proposed as a reversible drug tolerant state in individual tumor cells [93]. Some cancer cells are addicted to ecDNA, as it is a carrier to maintain oncogene amplification, which has the advantage of altering of their genomes at faster rates. It has been reported that CRC patients who suffered cancer cells with ecDNA have shorter survival than others [7].

Several studies have shown that alterations in the frequency of ecDNAs in multiply types of cancers after drug treatment [12]. Treatments may result in an elevation of the ecDNA copy number to promote acquired drug resistance, while some drugs may result in the diminishing of the ecDNAs harboring the target gene to decrease the sensitivity. For example, MTX is a chemotherapy drug for CRC that targets dihydrofolate reductase, which is the enzyme for nucleotide metabolism. However, CRC cells often develop resistance to MTX owing to amplification of dihydrofolate reductase gene (DHFR). Studies have shown that methotrexate treatment in CRC cells lead to increase in ecDNAs that contains DHFR leading to resistance to methotrexate [20, 94]. Following studies shows that withdrawing of MTX decreases ecDNA-mediated DHFR amplification in drug resistant CRC cells, and lack of the DHFR amplicon can reduce their ability to generate resistance [94]. Thus, CRC cells exhibit a reduction in the capacity to generate resistance for a second cycle of MTX administration. This evidences suggested a potential chemotherapy strategy to overcome drug resistance in CRC promoted by ecDNA-mediated amplification. In addition, the rise of drug targets on ecDNA, such as DHFR, MRP5, ATM, and P53, leads to better cell fitness under selection pressure [95]. Meng et al. revealed that the depletion of nonhomologous end joining proteins (NHEJ) in CRC cells leads to decreased amplification of DHFR, disappearance of ecDNAs, increased micronuclei formation and is correlated with the elimination of DHFR, thus increasing sensitivity to MTX [21]. It indicates that NHEJ plays a specific role in ecDNA formation and is presented as a promising target for CRC chemotherapy. It has also been demonstrated that homologous recombination activity was elevated in MTX resistant CRC cells. The silencing of the BRCA1 gene decreased the frequency of ecDNA and repressed the expression of amplified oncogenes [70]. Furthermore, BRCA1 depletion leads to MTX resistant CRC cells sensitive to MTX. Therefore, in CRC, the homologous recombination pathway could also serve as a potential target for therapies through the depressing of ecDNA-mediated oncogenic amplification. Song et al. investigated another example of ecDNA-mediated drug resistance in the BRAF mutant cell line. The results indicate that with increasing doses of BRAF and MEK inhibitors, ecDNA dramatically increased that arose BRAF copy number [57]. The ecDNA can reintegrate into the chromosome with the aggregated HSR-like structure after continued drug treatment. Continue culture the drug-addicted cells with low concentration or in the absence of drug leading to a shortening or loss of HSR [57]. Rapid acquired resistance to treatment, driven by the dramatic plasticity of the genome engendered by ecDNA presumably plays an essential role.

In contrast, the amplification of the EGFR gene through ecDNA is frequently observed in cancer cells and is associated with invasiveness, heterogeneity, and radioresistance [41]. However, it is still controversial that eliminating EGFR containing ecDNA to downregulate gene expression alleviates these malignant phenotypes. Acquiring drug resistance in cancer patients could due to time consuming selection of drug sensitive mutations, such as oncogenic variant EGFRvIII, which accelerates tumor proliferation but results in cancer cells more sensitive to tyrosine kinase inhibitors (TKIs), declining when cells become drug resistant [6]. Staining of HSRs indicates that these ecDNAs reintegrated into chromosomes and that resistance to cancer cells occurs after the elimination of EGFRvIII positive ecDNA. After withdrawing the inhibitor, the reemergence of EGFRvIII amplification in ecDNA [6]. Oncogenic ecDNAs are not just limited to EGFR variants and are likely to apply to most oncogenes across different types of cancer. Following the successes with TKI, such as the anti-EGFR agent cetuximab and the anti-angiogenesis agent bevacizumab, these mechanisms could play an important role in the target therapy in CRC. Thus, ecDNA oncogene amplification could serve as predictive biomarkers for therapies, and the implication of ecDNAs for drug resistance places it as one of the most important targets for future therapy.

## Target ecDNA for colorectal cancer therapy

### Target for the biogenesis of ecDNA

**Fig. 3:**
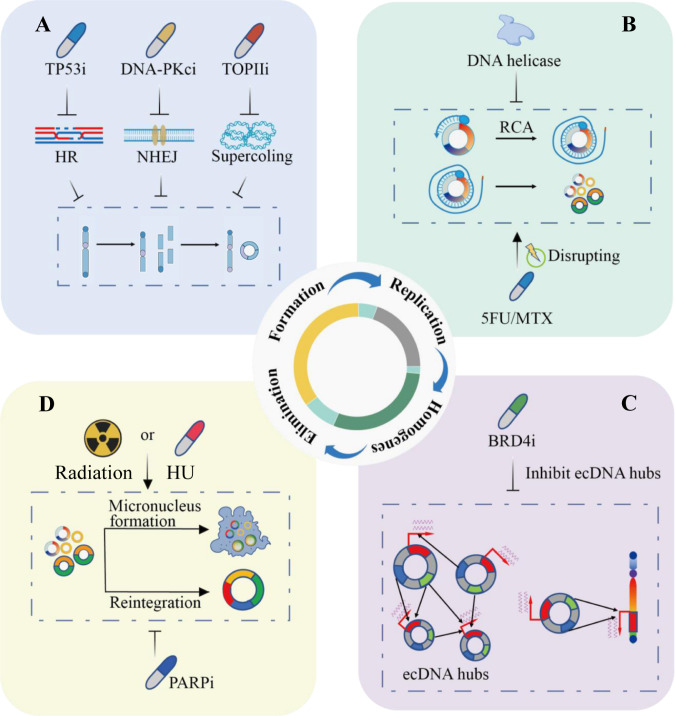
Target ecDNA for colorectal cancer therapy. **A** Targeting at biogenesis of ecDNA. The inhibitors of TP53, DNA-PKcs, and TOPII can repress ecDNA formation by inhibiting homologous recombination (HR), nonhomologous terminal ligation (NHEJ), and DNA supercoiling respectively. **B** Targeting at ecDNA replication and distribution. DNA helicase inhibits rolling circle amplification to suppress the replication process of ecDNA, 5-FU and MTX affect ecDNA replication by directly destroying ecDNA. **C** Targeting at ecDNA hub. BRD4 inhibitors disrupt aggregation of ecDNA hubs, thus disturbing ecDNA intermolecular regulation and interactions between ecDNA and genomic DNA. **D** Targeting at elimination of ecDNA. Radiotherapy and HU can reduce ecDNA frequency by promoting the formation of micronuclei, and PARP inhibitors eliminate ecDNA by promoting ecDNA fusion.

ecDNA can be found in tumor samples from patients with various types of cancer [7]. Up to now there has been still a lack of biomarkers that can be detected for the biogenesis of ecDNA in CRC patients, and less is known about potential therapeutic and diagnostic targets of ecDNA. Potential targets could be postulated to involve enzymatic activities in ecDNA genesis or repression of amplified oncogene expression. The sequences of the circle junction in ecDNAs may also be taken as drug targets. Repressing the ecDNA biogenesis might serve as a potential strategy for CRC patients. In CRC cells, DNA repair following chromothripsis, as well as other DNA breakage activities, could lead to the formation of ecDNA [37]. Chromothripsis is more frequently found in chromosomally unstable cells such as MSI-H CRC, which accompany with higher rates segregation errors of the chromosome or with p53 checkpoint deficient. Therefore, TP53 is a predisposing factor for chromothripsis. In addition, disruption of DNA damage repair processes, such as homologous recombination, by PARP inhibitors could suppress the frequency of ecDNA, and therefore may be a potential therapeutic target (Fig. 3A). Meanwhile, NHEJ during DNA damage repair has been shown to be involved in ecDNA formation in CRC cells, and DNA dependent protein kinase inhibitors could disrupt this process [21]. And DNA-PKcs inhibitors may disadvantage tumors that benefit from ecDNA amplifications (Fig. 3A). The frequency of ecDNA is dramatically reduced after treatment [37]. This strategy could be more effective in combination with other DNA damaging agents, as well as radiotherapy. Additionally, DNA supercoiling occurs during transcription which needs to be resolved by topoisomerases. Topoisomerase II will also result in double-strand breaks during the DNA damage repair process that ecDNAs may be generated. The overlap of ecDNAs and RNA polymerase also implies high topoisomerase II activity that can form double-strand breaks as a mechanism for reintegration into the linear chromosome and other ecDNA moleculars [96] (Fig. 3A).

### Targeting in ecDNA replication and distribution

DNA replication initials with unwinding of the double helix by DNA helicases. A large number of helicases show various specific functions in DNA repair pathways and keep genomic stability. Some drugs have been applied to the clinical treatment of CRC that modulate helicase expression or functions, which is a viable approach to inhibit cancer cells through the inactivation of DNA replication restart, helicase-dependent DNA repair pathways, and cell cycle checkpoint [97] (Fig. 3B). However, the replication of ecDNA could be subject to a specific helicase, as occurs in mitochondrial DNA replication [98]. It will be interested to know which types of helicase inhibitors will repress the formation or replication of ecDNA in CRC. Another option is to disrupt the building blocks of DNA, deoxyribonucleotide triphosphate (dNTP), which is commonly used as the target for chemotherapy. In CRC, first-line chemotherapy includes nucleoside synthetase inhibitors such as 5-Fu and capecitabine, ribonucleotide reductase inhibitors hydroxyurea and gemcitabine, as well as the dihydrofolate reductase inhibitor methotrexate (Fig. 3B). All these chemotherapy agents may to some extent interfere with ecDNA replication. Further studies may focus on whether there are any ecDNA-specific replication enzymes. Following replication, ecDNAs could then segregate into daughter cells through hitchhiking on chromosomal DNA [67]. The mechanism of ecDNA to attach to chromosomes and the molecular glue that may exist remain elusive. A deeper understanding of ecDNA segregation may provide opportunities to modulate the process.

### Targeting in the ecDNA hub

The spatial distribution of ecDNA, which could generate ecDNA hubs, can lead to trans-interactions between intermolecular ecDNAs or ecDNAs and chromosomal DNA. Dispersal of ecDNA hubs was correlated with reduced expression of oncogenes in ecDNA-positive cancer cells. Thus, the abnormal distribution of ecDNA may represent a vulnerable target and this unique ecDNA function can be pharmacologically perturbed (Fig. 3C). Factors involved in ecDNA hubs could be considered as potential therapeutic targets to repress the transcription and activity of the ecDNA cargo [80]. For example, the stability of ecDNA hubs has been demonstrated to be associated with the existence of the BET protein BRD4 [55, 99]. The ecDNA hubs are tethered by the bromodomain and the BET protein BRD4 in a MYC-amplified CRC cell line. In CRC cells, it has been studied that dispersal of MYC ecDNA hubs through inhibition of the and BET proteins, the inhibitor JQ1 disrupts ecDNA the hubs and represses ecDNA derived oncogene transcription [80]. Targeting the BET protein BRD4, which could be applicable in disturbing the ecDNA tethering [80]. More studies are required to determine whether BRD4 plays a decisive role in maintaining the stability of ecDNA hubs in CRC and if other factors also play similar roles. For instance, long noncoding RNAs (lncRNAs) are also shown to be involved in the formation of interchromosomal interactions [100]. CRISPR interference (CRISPRi) has been applied to target gene promoters on ecDNA, as well as noncoding regulatory DNA elements, including enhancers [9, 47]. CRISPRi disruption of enhancers by has identified enhancers that elevated oncogene expression on ecDNAs that increase ecDNA-positive cancer cell survival. CRISPRi can silence ecDNA promoters despite elevated copy numbers potentially due to combinatorial interactions between enhancer and gene interplays or compensation by other enhancers within ecDNA hubs [80]. These studies indicate that an enhancer hijacking mechanism and intermolecular cooperativity in ecDNA hubs could potentially be regulatory elements for ecDNA function in CRC. Treatment of CRC cells by interfering with the formation of ecDNA hubs is a promising target, but there are still some aspects that need to be elucidated.

### Targeting at elimination of ecDNA

Tumor heterogeneity is a critical reason for acquired drug resistance. ecDNA reintegration leads to genome remodeling that affects gene expression [9]. Reintegration of ecDNA can also exert some adverse effects, for example, disrupting of tumor suppressor genes [4], affecting the oncogene expression [37], and inducing genomic instability [101]. Additionally, ecDNA fragments may also be integrated into the upstream of proto-oncogenes to enhance the expression of proto-oncogenes. On the other hand, ecDNA fragments may also be inserted into suppressor genes, resulting in loss of tumor suppressor gene function and promoting tumor development and development [4]. PARP has been shown to decrease the frequency of ecDNA reintegration, thus being a potential therapeutic target [37] (Fig. 3D). Furthermore, ecDNA is more prone to micronuclear expulsion and elimination than linear chromosomal DNA; thus, the formation of micronuclei is due to ecDNA elimination [102]. It has been shown that the antimetabolite hydroxyurea (HU), an inhibitor of DNA replication, can eliminate ecDNA and reduce tumorigenicity and increase drug sensitivity [103, 104]. Although HU does not present good clinical antitumor activity in CRC, either HU is not used in the treatment of ecDNA-positive tumors, these studies provide the theoretical basis for further drugs screening [105]. In addition to chemotherapy, Schoenlein et al has shown that reduction in ecDNA, amplified gene copy number, and expression level was detected in surviving CRC cells after radiation exposure [106]. Radiation therapy could be able to entrap ecDNA that carries drug resistance genes MDR1 and MYCC oncogenes in radiation-induced micronuclei [106, 107] (Fig. 3D). Micronucleation could repress DNA repair of entrapped ecDNAs, therefore, rendering ecDNA vulnerable to DNA damaging strategies. Further in-depth studies and analysis of the molecular signal pathways involved in micronuclear expulsion could reveal novel therapeutic targets.

## Conclusions and future perspective

The genome of CRC is not static, but dynamic and the function of ecDNAs could be attribute to their size as well as genes or regulatory elements. The characteristics and mapping of ecDNA updated many fundamentals of what we understand about various types of cancer, including CRC. Initially, oncogene amplification on ecDNA is a common driving force for tumor growth, as well as resistance to drug, leading to a worse prognosis. Secondly, ecDNAs provide an accelerated tool for heterogeneity and the genomic evolution of CRC. Additionally, gene expression of oncogenes on ecDNAs is more complicated because of the structure and dynamics of ecDNA. Circular structures and the formation of ecDNA hubs brought together in the trans regulation of chromosomal enhancers across different ecDNA molecules. The application of current molecular analysis to ecDNAs reveals new fundamentals about the remodeling and evolution of the cancer genome. ecDNA attributes to the three pillars of Darwinian evolution (inheritance, variation, and selection) in ways that vary from contributions from linear chromosomes.

### Potential and challenges of ecDNA as a diagnostic and therapeutic target for CRC

Further breakthroughs in ecDNA biology require efforts in genomic technology development. Despite its prevalence, ecDNA detection is limited by the specificity, sensitivity, and resolution of current methods [22]. Live cell imaging-based methods can distinguish chromosomal and ecDNA signals, but suffer from limited throughput and also DNA sequence-dependent. To profile the landscapes, ecDNAs have to be isolated from cancer cells for targeted profiling and comparisons between ecDNAs and chromosomes. Circle-seq was developed for unbiased isolation of circle DNA [4]. CRISPR–Cas9-assisted targeting of chromosome segments (CRISPR–CATCH) allows the separation of linear chromosomal DNA and ecDNA from the same sample for direct comparisons, as well as enables the profiling of the genetic sequence of ecDNA and the epigenomic landscape [108]. High-throughput sequencings such as WGS or Circle-seq enabled detailed characterization of the cancer genome and epigenome. However, oncogene copy-number amplifications or whole-genome sequencing have limitations for ecDNAs detection of clinical tumor samples. Further studies required with novel single cell-based methods expected to provide novel insights into ecDNA intercellular heterogeneity and structure–function relationships. Development of empirical ecDNA-detection strategies from clinical samples is needed, which detects sequence and structural features of ecDNAs in tumors, thus systematic evaluation of clinical outcomes associated with ecDNAs.

As a unique molecular feature for the tumor, ecDNAs become potential diagnostic biomarkers and therapeutic targets in CRC. ecDNAs in circulating cell-free DNA (cfDNA), which has been explored for the non-invasive diagnosis and management of tumors detected in plasma and serum, have been identified and investigated as diagnostic markers [109]. It also reported that ecDNAs have been identified in urine and that multiple types of ecDNA could be applied as diagnostic markers for early detection of CRC [110, 111]. The circular structure of ecDNAs makes them less susceptible to exonucleases compared with linear cfDNAs. Although some of the studies focus on the smaller size of ecDNA, the above studies indicate that circular DNA can be released into circulation. Progress has been made in characterizing the chromatin composition and the unique sequence of ecDNAs. Further studies are required that examine ecDNAs as biomarkers in CRC, to evaluate specificity and sensitivity. The future directions in the field are to develop routine ecDNA detection in the clinic and enable improved molecular stratification of patients with CRC in clinical trials to increase the translational potential of novel targeted therapies.

It is exciting time, as the field of ecDNA research in various types of cancer, including CRC, is making huge progress with the help of advanced technologies as well as multidisciplinary collaborations. At the same time, several challenges were encountered as a therapeutic target or diagnostic marker. ecDNA represents a diverse group of focal amplifications, which are different in size and structural complexity. The heterogeneity suggested that different strategies are required to target each subclass of ecDNA. Secondly, we currently have limited data on the capacity of ecDNAs to reintegrate into the genome and diminish in CRC cells [112]. For instance, reintegration is a mechanism of drug resistance due to the reemerge extrachromosomally after the drug is removed [6]. Meanwhile, the presence of ecDNA in CRC has been widely shown, however, we lack understanding of the clonality of ecDNA. Especially lack of clinical data for observation of CRC patients during chemo or target therapies. Finally, passing through multiple cellular membranes to target a molecule in the nucleus is always challenging. As various mechanisms are involved in the biogenesis, maintenance and evolution of ecDNA, targeting at any individual step may not be enough to successfully treat ecDNA-positive CRC. Moreover, given the complex and heterogeneity of each subcategory of CRC, such as MSS, MSI-L, and MSI-H, it is impossible to find a one-size-fits-all approach. However, it is believed that lowering the frequency of ecDNA could reduce the risk of resistance to ecDNA-driven therapy, and improve therapy efficiency.

### Opening questions

There remain open questions such as whether the biogenesis of ecDNA is a result of selection pressure for transcriptionally active molecules or is it inherent between circular extrachromosomal chromatin and native chromosome? Is there a specific composition of ecDNA hubs in the CRC for each subcategory? Is the preference for ecDNA segregation in the form of singletons or smaller hubs in CRC? Whether the spatial distribution of ecDNA hubs is random or directed, and will the composition change with passage of cells? ecDNAs were discovered more than half a century ago; nevertheless, their prevalence and the central role in cancer, as well as CRC pathogenicity, are only just beginning to be appreciated. New tools and methods to investigate the characteristic and spatial organization of the genomes of ecDNA-positive cancers provide novel insights into their biology and enable the development of clinical intervention strategies targeting ecDNA. In conclusion, ecDNA may be a potential target for diagnosis, treatment, and prognosis in CRC.

## Data Availability

Data availability is not applicable to this article as no new data were created or analyzed in this study.
